# Weight Change over Ten Years Predicts Biological Aging in a Random Sample of 3070 U.S. Adults

**DOI:** 10.3390/nu15132862

**Published:** 2023-06-24

**Authors:** Larry A. Tucker, Joshua A. Brockbank

**Affiliations:** 1 College of Life Sciences, Brigham Young University, Provo, UT 84602, USA; 2 School of Family Life, Human Development, Brigham Young University, Provo, UT 84602, USA; josh.brockbank@gmail.com

**Keywords:** obesity, weight gain, telomere, DNA, disease, inflammation, CRP

## Abstract

This investigation was designed to study the relationship between weight change over 10 years and leukocyte telomere length (LTL) in a large sample of 3070 randomly selected U.S. adults, 36–70 years old. The National Health and Nutrition Examination Survey (NHANES) data were used to examine the relationship between percent weight change and LTL. Potential mediating variables were controlled using partial correlation. After adjusting for age, race, year, and housing status, the association between percent weight change over 10 years and LTL was significant in women (F = 6.9, *p* = 0.0138). Adjusting for the demographic and several other covariates weakened the relationship slightly (F = 4.7, *p* = 0.0392). With all the covariates controlled, for each one percentage point increase in weight over the previous 10 years, telomeres were, on average, 3.48 base pairs (bp) shorter in women. Given that each one-year increase in age was associated with telomeres that were 15.0 bp shorter in women, the median weight change in U.S. women over the previous 10 years (an increase of 10.4%) was predictive of LTLs that were 36 bp shorter, on average, or an increase of 2.4 years of biological aging. Percent weight change over 10 years was not associated with LTL in U.S. men. Percent weight change over 10 years is a strong predictor of biological aging in U.S. women, but not in men.

## 1. Introduction

Telomeres play vital roles in preserving our genomes from degradation and protecting chromosomal ends from fusion. Telomeres are structures at the end of chromosomes comprised of nucleotide repeats. During DNA replication in the absence of telomerase, telomeres shorten with each division due to incomplete replication of the lagging strand by the DNA polymerases. Therefore, as the chronological age of humans increases, the length of telomeres generally shortens [1,2].

Many factors influence telomere length, some of which include stress [3], diet [4,5,6], physical activity [7,8], and obesity [9]. While telomere shortening is a natural process, short telomeres are predictive of increased risk of cardiovascular diseases [10,11], cancer [12,13], insulin resistance [14,15], and early mortality [16]. When telomeres become too short, the chromosome reaches a critical length and can no longer replicate, which leads to cellular senescence.

Obesity leads to many health disorders. Excess body weight increases the risk of developing type 2 diabetes [17], cancer [18], cardiovascular diseases [19], and other age-related disorders [20]. With rates of obesity increasing across the population, understanding its effect on health and well-being is important. In individuals with obesity, high oxidative stress and systemic inflammation are observed [21]. These have been shown to shorten telomere lengths.

Multiple studies have focused on the association between telomere length and obesity [9,22], and a few studies have examined weight change over time [23,24,25]. However, more research in this area is needed because previous investigations have produced inconsistent results and absolute weight change may not be the best outcome to study. For example, a change in mass of 10 kg for a 115 kg person is quite different than a 10 kg change in a 65 kg person. In addition, reporting change in BMI units does not reveal how much weight has been lost or gained. Thus, measuring percent weight change instead of absolute weight change or change in BMI may be a more appropriate and useful variable to compare with telomere length. Recent weight management guidelines support the use of percent weight change over absolute weight change [26]. Following this strategy may help to clarify the effect weight change has on the length of telomeres and biological aging.

The purpose of this study was to analyze the relationship between percent weight change over 10 years and telomere length in a large sample of 3070 randomly selected U.S. adults, 36–70 years old. Covariates, including age, race/ethnicity, year of assessment, housing status, intent to lose weight, BMI, pack-years of smoking, total physical activity, C-reactive protein, and disease status (i.e., having or not having diabetes, cardiovascular disease, and/or cancer) in men, and these covariates plus menopause status and hormone use in women, were controlled statistically to help isolate the relationship between percent weight change and telomere length. The associations were studied separately in men and in women.

## 2. Materials and Methods

### 2.1. Study Design and Sample

The National Health and Nutrition Examination Survey (NHANES) uses a multistage random sampling technique to collect information about the health and nutritional status of individuals in the United States to represent the nation’s population. Data for this study were obtained from two 2-year cycles (1999–2000 and 2001–2002). Although the NHANES data have been collected for decades, these two 2-year ranges are the only years that HANES collected data on telomere length, which is why these two cycles were used in this study. The NHANES data are available to everyone online without cost for those seeking health and nutrition information representing the United States population [27,28].

The sample for this study included individuals who were 36–70 years old because the exposure variable, i.e., 10-year weight change, required body weight from 10 years previous to be assessed. Those younger than 36 years old were excluded because there is still natural physical maturation resulting in normal increased body mass in the early twenties. The youngest individuals included in the study were 26 years old at the beginning of the 10-year period. At age 26, the human body is fully developed. Those over the age of 70 were excluded because body weight problems associated with old age, particularly frailty and serious diseases, are common. Individuals with extreme weight loss over the previous 10 years (more than 3 standard deviations) were not included in the sample. Pregnant women were also excluded.

### 2.2. Weight Change

Current body weight was measured directly by HANES, and body weight 10 years earlier was self-reported by participants. The variable 10-year percent weight change was calculated by subtracting the initial weight 10 years earlier from the current body weight, and then dividing that number by the initial body weight. This value was then expressed as a percentage.

Several quality studies have indicated that self-reported body weight is an accurate source of measurement for actual body weight [29,30,31,32,33]. According to Phimphasone-Brady et al., “Self-report and objective weight assessments showed high concurrent validity” [29]. Additional research has shown correlations of greater than or equal to 0.98 between self-reported and observed body weight [30,31,34]. An investigation of 1302 adults by Stunkard et al. [30], published in the *American Journal of Clinical Nutrition*, showed a high correlation between self-reported body weight and measured weight in U.S. adults (*r*  =  0.99, *p* < 0.01), where Stunkard concluded [30], “Self-reported weights were remarkably accurate across all these variables in the American sample, even among obese people, and may obviate the need for measured weights in epidemiological investigations” [P. 1593].

In an investigation that focused on a homogeneous sample of 355 Polish men, 35–80 years old, participants, on average, overestimated their height by 1.0 cm and underestimated their weight by 0.9 kg [35]. Additional studies have shown high accuracy of long-term weight recollection [36,37]. Adults, aged 71–76, who recalled their heights and weights during their last year of high school were in close agreement with the actual values [36], which supports that self-reported recalled weight from 10 years earlier is valid. According to Willett, recalled values and measured BMIs from approximately 55 years later had a correlation of 0.91 (*p* < 0.01) for men and 0.92 (*p* < 0.01) for women, stating that differences between recalled and actual values “have minimal effect on epidemiologic measures of association” (P. 247).

In another study of 1805 U.S. men, Rhoads et al. found a difference of only 2.2% between recalled and measured draft registration weight at age 25 when they were 20–30 years older [38]. In the same study, a sample of 118 women, 25–42 years old, were asked to recall their weight at age 18. The weight difference was 1.4 kg between recalled and actual weight based on physical examination at age 18, and the correlation was 0.87 (*p* < 0.01). 

### 2.3. Covariates

In the present investigation, covariates of age, race, year of assessment, housing status, BMI, intent to lose weight, smoking, total physical activity, C-reactive protein, and disease status (i.e., having or not having diabetes, cardiovascular disease, and/or cancer) were controlled statistically in men. Two additional covariates, i.e., menopause status and hormone use, were also assessed and controlled statistically in women. Age was self-reported by participants. HANES reported sex as either male or female. Race was defined by NHANES as non-Hispanic White, non-Hispanic Black, Mexican American, other Hispanic, and other race/multiracial. Housing status was assessed by participants reporting their housing situation, i.e., renting, buying, or other. 

BMI was calculated by dividing body weight in kg by height in meters, squared. Underweight was defined as a BMI < 18.5. Underweight individuals were not included in the sample because of the increased likelihood of having a serious disease or an eating disorder. Normal weight was defined as a BMI of 18.5–24.9; overweight was defined as a BMI of 25.0–29.9; obesity was defined as a BMI of 30.0 or higher. 

Smoking was quantified by using pack-years. Pack-years of smoking were calculated by multiplying the number of cigarettes smoked per day by the number of years the person smoked, divided by 20 (the number of cigarettes in a pack). Pack-years were divided into four categories. The vast majority of subjects reported never smoking (0 pack-years). The remaining participants were divided into sex-specific tertiles. For men, there were four pack-year categories, i.e., 0, 0.1–9.9, 10.0–23.4, and >23.4 years. For women, the pack-year categories were 0, 0.1–2.7, 2.8–20, and >20.0.

To assess physical activity (PA), metabolic equivalent (MET) minutes were used. The metabolic equivalent is the amount of energy expended at rest. Participants reported the number of days per week and the amount of time per session, if any, of 48 physical activities they engaged in over the past 30 days and whether the activity was moderate or vigorous in intensity. HANES provided examples to help participants understand the differences between moderate and vigorous PA. A MET value for each activity was assigned using the compendium of physical activity [39]. Total physical activity was calculated by summing the MET minutes of each activity and converting this score to a weekly value.

Because natural menopause and some medical interventions (e.g., hysterectomy, oophorectomy, etc.) cause cessation of menses and can influence the health of women, they were controlled in the present study. Self-reporting was employed to gather the data. Using the information, women were divided into two groups, i.e., those who no longer menstruated because of menopause or medical treatments and those who continued to have periods.

Hormone use was also assessed in female participants via self-reporting. Two categories were formed, i.e., women who currently use estrogen and/or progesterone or have in the past, and women who have not used estrogen and/or progesterone. Hormone use for birth control was not included in the assessment.

### 2.4. Telomere Length

In the present study, the quantitative polymerase chain reaction (qPCR) method was employed to measure telomere length relative to standard reference DNA, as described elsewhere [40,41]. qPCR is a cost-effective process that can be conducted by using small amounts of DNA available from stored samples. The qPCR approach is excellent for big epidemiological investigations that involve thousands of samples. Potential irregularities across various components of the qPCR telomere length assay emphasize the need for careful and precise measurement methods across each step of the process. Because of this, NHANES contracted with the University of California, San Francisco (UCSF) to perform the telomere length measurements.

The UCSF lab included many high-level quality-control checks to ensure valid and reliable results. According to NHANES, “Each sample was assayed 3 times on 3 different days. The samples were assayed on duplicate wells, resulting in 6 data points. Sample plates were assayed in groups of 3 plates, and no 2 plates were grouped together more than once. Each assay plate contained 96 control wells with 8 control DNA samples. Assay runs with 8 or more invalid control wells were excluded from further analysis (<1% of runs). Control DNA values were used to normalize between-run variability. Runs with more than 4 control DNA values falling outside 2.5 standard deviations from the mean for all assay runs were excluded from further analysis (<6% of runs). For each sample, any potential outliers were identified and excluded from the calculations (<2% of samples). The mean and standard deviation of the T/S ratio were then calculated normally. The interassay coefficient of variation was 6.5%” [42]. To transform T/S ratios to base pairs, the following equation was employed: 3274 + 2413 × (T/S).

### 2.5. Data Analysis

A multi-level, probability, sampling strategy was employed by HANES to randomly select participants from the United States population. A total of 28 strata followed by 57 clusters were randomly selected. Each participant was assigned an individual sample weight by NHANES based on census data. Unbiased national estimates resulted because the strata, clusters, and sample weights were utilized in each statistical analysis. In other words, the results can be generalized to the adult, civilian population of the USA because of the stratified random sampling technique employed to collect the data. In the full statistical model, there were 29 degrees of freedom in the denominator (57 clusters minus 28 strata). Statistical significance was based on the 29 degrees of freedom, not the 3000+ participants in the investigation.

Means and standard errors (±SE) were used to summarize continuous variables, and frequencies with standard errors were employed to summarize categorical variables. A regression analysis using the SAS SURVEYREG procedure along with strata, clusters, and individual sample weights, was employed to measure the magnitude of the linear associations between weight change over the previous 10 years and telomere length, each treated as a continuous variable. To highlight the extent that the lengths of telomeres varied according to differences in weight change over the previous 10 years, regression coefficients were reported. Because telomere lengths and weight change over the previous 10 years both differed between men and women, analyses were performed separately within each sex. To control for differences in the covariates (age, race, housing status, intent to lose weight, body mass index, smoking pack-years, total MET minutes of physical activity, C-reactive protein, and disease status in men, and these covariates plus menopause status and hormone use in women), partial correlation was used. 

To perform the statistical analyses, the SAS version 9.4 (SAS Institute, Inc., Cary, NC, USA) software was used. All *p*-values were two-sided and *p* < 0.05 was the standard set to determine statistical significance.

## 3. Results

The NHANES sample representing the U.S. population included 3070 participants. The mean age (±SE) of the sample was 50.2 ± 0.3 years and the average telomere length was 5777 ± 41 base pairs. The mean weight change over the previous 10 years was 7.9 ± 0.3 percent.

Table 1 shows the median and percentile values for age, percent weight change, telomere length, and C-reactive protein levels for women and men representing the U.S. adult population. The table is divided by sex. On average (mean ± SE), men increased 5.1 ± 0.3 percent in body weight while women increased 10.8 ± 0.4 percent over the previous 10 years.

Table 2 shows the number and percentage of the 3070 subjects for each of the categorical variables of the investigation. Overall, there was about an equal number of women and men. Moreover, participants tended to be non-Hispanic White, buying their residence, non-smokers, physically inactive, overweight, with no intent to lose weight, and with no serious disease. About two-thirds of the female sample were no longer having periods and approximately the same number were currently using estrogen and/or progesterone or they had used these in the past. 

Table 3 shows the relationships between weight change over the previous 10 years and telomere length in women and men viewed separately, after adjusting for all the covariates. The relationship between each covariate and telomere length is also shown, with all the other variables in the table controlled. Not shown in the table was the finding that after adjusting for differences in the demographic covariates only (age, race, year of assessment, and housing status), weight change over the previous 10 years was a significant predictor of the length of telomeres in women (F = 6.9, *p* = 0.0138). Specifically, for each 1 percentage point increase in 10-year body weight, telomeres were 4.41 base pairs shorter, on average. As shown in Table 3, with all the covariates controlled, for each 1 percentage point increase in 10-year weight change, telomeres were 3.48 base pairs shorter, on average (F = 4.7, *p* = 0.0392). However, the percent weight change over 10 years was not related to telomere length in men with the demographic covariables controlled (F = 1.0, *p* = 0.3331), or after adjusting for all the covariates (F = 1.6, *p* = 0.2099).

Not displayed in a table, the difference in 10-year weight change between women and men was significant (F = 175.9, *p* < *0*.0001) with age, race, year of assessment, and housing status controlled statistically. Likewise, with the same demographic variables controlled, men and women tended to differ in telomere length. Specifically, men (mean ± SE) 5722 ± 51 had shorter telomeres than women 5780 ± 49 (F = 3.8, *p* = 0.0609). Additionally, in women, the relationship between age and telomere length was strong and significant (F = 66.2, *p* < *0*.0001). Specifically, for each one year of chronological age, telomeres were, on average, 15.0 base pairs shorter. In men, age and telomere length were also strongly and significantly related (F = 124.9, *p* < *0*.0001), and for each year of chronological age, telomeres were, on average, 16.7 base pairs shorter. 

## 4. Discussions

The focus of this study was to examine the relationship between percent change in body weight over 10 years and leukocyte telomere length in a large, nationally representative sample of U.S. adults, 36–70 years old. The results showed that as the percent weight change over the previous 10 years increased, the length of telomeres were shorter in women, but not in men. Another purpose was to evaluate the weight change and telomere relationship after adjusting statistically for a number of potentially confounding covariates.

Key findings showed that for women, with age, race, housing status, and year of assessment controlled statistically, each percentage point of weight increase over 10 years was associated with telomeres that were 4.41 base pairs shorter, on average (F = 6.9, *p* < 0.0138). Even with all the covariates factored in, there was a meaningful and significant relationship with telomeres being, on average, 3.48 base pairs shorter for each percentage point of weight change (F = 4.7, *p* = 0.0392). 

A possible factor accounting for why the association for men was not significant is that the percent weight gain over the previous 10 years was substantially lower in U.S. men than women. Specifically, half the men (50th percentile) gained 5.2% or more in body weight over the previous 10-year period, whereas half the women gained 10.4% or more, i.e., twice the increase of the men. Further differences were shown between genders at the 75th percentile, where U.S. men gained 11.9% in weight and U.S. women gained 19.3%. Because relationships are difficult to detect when variation is limited, and because the weight gain distribution was much greater in U.S. women than men, initially and 10 years later, these factors could account for some of the relationship between weight change and telomere length identified in women but not in men.

Weight gain is a process, whereas obesity is a result of that process. Therefore, weight gain and obesity are significantly related. Weight gain and obesity patterns vary across cultures, races, and nations. Within the USA, from 1960–2016, women had higher rates of obesity and severe obesity each year compared to men [43]. Women would not have had higher rates of obesity and severe obesity than men unless they gained more weight across time than men. 

In a recent investigation of insured U.S. adults (*n* > 115,000) by Buszkiewicz et al., with follow-ups at 1, 3, and 5 years, women gained more weight than men and the difference in weight gain increased across the follow-up periods [44]. Likewise, in a study of almost 14,000 randomly selected U.S. adults from 2011–2018, women gained significantly more weight than men over time [45]. The findings of these investigations support the results uncovered in the present study showing that U.S. women tend to gain more weight over time than U.S. men. 

Interpretation of the telomere results of this study can be expressed as differences in biological aging based on weight changes. Step 1 of the interpretation begins with the fact that, in U.S. women, each year of chronological age was associated with telomeres that were 15.0 base pairs shorter. Step 2 includes the point that for each percent body weight increase over 10 years, telomeres were 3.48 to 4.41 base pairs shorter, depending on the covariates controlled. Step 3 includes the finding that the 50th percentile for women increased 10.4 percent in body weight over the previous 10 years. Step 4 combines these data resulting in a biological aging outcome of approximately 2.4 to 3.1 years, depending on the covariates controlled. This means that U.S. women at the 50th percentile for weight change over 10 years tended to be about 2.4 to 3.1 years older biologically than women who did not gain any weight over the previous decade (10.4 × 3.48 = 36.2 ÷ 15.0 = 2.4) and (10.4 × 4.41 = 45.9 ÷ 15.0 = 3.1). This accelerated aging was further demonstrated by using the 75th percentile for women, which increased 19.3% in body weight. The 19.3% weight gain was associated with being approximately 4.5 to 5.7 biological years older compared to women who did not gain any weight (19.3 × 3.48 = 67.2 ÷ 15.0 = 4.5) and (19.3 × 4.41 = 85.1 ÷ 15.0 = 5.7). 

In comparison, another study has shown that adults reporting 25 pack-years of smoking have an estimated 4.6 years of increased cellular aging compared to non-smokers, which is less biological aging than the 75th percentile in weight gain for women in the present study. Consumption of sugar-sweetened soda appears to increase cellular aging by 1.8 years for each 8 ounce serving per day [46]. Furthermore, research shows that for each 100 mg of caffeine consumed per day, adults have approximately 2.3 years of increased biological aging [47]. Hence, average percent weight gain over 10 years in U.S. women appears to be predictive of substantial increases in biological aging whether considered absolutely or relative to other lifestyle factors. 

Other studies have investigated the relationship between weight change and telomere length [23,24,25]. However, the present study aimed to clarify inconsistent results and to strengthen limitations of previous investigations. Zhang et al. found significant relationships between obesity and weight gain and telomere length. Their data showed that adults with stable obesity had telomeres that were 0.130 kilobase pair (95% CI 0.061–0.198, *p* = 0.0002) shorter than those with stable normal weight. Their study also found that weight gain from the non-obesity category to the obese category was predictive of shorter telomeres by 0.094 kilobase pairs (95% CI 0.012–0.177, *p* = 0.026), and moving from the normal weight category to the overweight category was associated with telomeres that were 0.074 kilobase pairs shorter (95% CI 0.014–0.134, *p* = 0.016) [23]. Movement between body weight categories is informative. However, weight change expressed as a percentage gives greater clarification and precision to the findings. Current weight change guidelines tend to focus on percent weight change [26].

Cui et al. also found an inverse relationship between weight gain and telomere length. Their study included 2912 Chinese women and they found that those who gained more than 15% weight had approximately 4% shorter telomeres than those who maintained their body weight within ±5% of their weight at age 50 [24].

In addition, Kim et al. found a significant relationship between change in BMI and telomere length. Their findings showed a linear decrease in mean relative telomere length with increasing BMI (*p* = 0.03). However, the sample included only women and the vast majority of the women gained weight and only a few women lost weight over time [25].

There appear to be underlying mechanisms for the association between percent weight change and telomere length. Elevated body weight and fat are known to increase body-wide inflammation and oxidative stress, which leads to shorter telomeres [21,48,49,50,51]. Therefore, C-reactive protein (CRP), a systemic measure of inflammation, was controlled in the current study. Across the distribution, men had significantly lower levels of C-reactive protein (CRP) compared to women. Moreover, after adjusting for differences in the demographic covariates, the relationship between CRP and telomere length in U.S. women was strong (F = 13.3, *p* = 0.0010), whereas in U.S. men the relationship was not significant (F = 3.5, *p* = 0.0701). In addition, with all the other variables controlled, CRP was a significant predictor of telomere length in women (F = 4.7, *p* = 0.0391), but was not significant in men (F = 1.4, *p* = 0.2541). Similarly, with the demographic covariates controlled, percent weight change was not associated with CRP in men (F = 0.09, *p* = 0.7691), but percent weight change was strongly related to CRP in women (F = 35.6, *p* < 0.0001). In short, body-wide inflammation appears to be a critical factor accounting for the relationship between percent weight change and biological aging in women, and seems to explain, in part, the percent weight change and telomere length relationship difference between men and women in the present investigation. 

Diet can also positively or negatively contribute to inflammation and oxidative stress, but individuals gaining weight typically have poor diets which lead to increased inflammation and oxidative stress [52,53].

Adjusting for differences in menopause status and female hormone use had almost no effect on the relationship between percent weight change and telomere length in the women participants. 

Elevated body weight also leads to reduced physical activity [54,55]. Studies have shown a strong relationship between physical activity and telomere length [7,8,56]. Specifically, less physical activity has been linked with shorter telomeres. Therefore, as weight gain increases, physical activity decreases and telomeres tend to shorten. 

Clearly, weight gain results in many unhealthy changes, behavioral and physiological, which likely influence the telomere relationships identified in the present investigation. 

The present study was subject to limitations that must be acknowledged. Due to the design employed, it is not possible to establish causality between weight change and telomere length. Moreover, the collection of covariate data, such as physical activity, at only one time period, poses limitations on accounting for time-varying confounders. In addition to physical activity, another example is yo-yo dieting. Weight loss, followed by weight gain, followed by weight loss again, could play a role in biological aging. Additionally, utilization of self-reported data for the initial body weight assessment could have introduced recall bias, although many investigations indicate that body weight recall is quite accurate. Finally, although the present study included a measure of physiological stress, i.e., C-reactive protein, it did not include an assessment of emotional stress. Stress plays an important role in accelerated aging and in the length of telomeres. 

This investigation also had many strengths. The generalizability of the findings from this study is supported by the random selection of a large sample of 3070 subjects from the noninstitutionalized, civilian adult population aged 36–70 years in the United States. Additionally, the use of objective methods with many quality control checks for the measurement of the telomere length variable enhanced the reliability and validity of the results. In addition, the variables not collected by self-reporting were measured by well-trained scientists not affiliated with this study, which minimized the potential for experimenter bias. Furthermore, weight change was expressed as percent weight change, which past studies have not utilized. Using percent weight change is consistent with present weight management guidelines, and it affords more meaningful insight into weight gains in small and large individuals.

In summary, a number of studies have been performed about obesity and weight gain and telomere length [9,22,23,24,25]. However, obesity is static whereas weight change over time is a dynamic measure. Additionally, using absolute weight gain (kg) may not be a good index because it lacks meaningful information about weight gain differences between normal weight and obese adults. Therefore, the present investigation focused on percent weight change over 10 years and its relationship with leukocyte telomere length in men and women representative of the U.S. adult population.

## 5. Conclusions

In conclusion, percent weight change over 10 years was a significant predictor of telomere length in U.S. women, but not in U.S. men. As 10-year percent weight gain increased, telomere length decreased. After adjusting for differences in all the covariates simultaneously, each percent weight gain over the previous 10 years was associated with a decrease of 3.48 telomere base pairs in women. Because each year of chronological age was associated with telomeres that were 15.0 base pairs shorter, the average woman in this study experienced an estimated 2.4 to 3.1 years in accelerated biological aging associated with her weight gain. Women at the 75th percentile of percent weight gain had approximately 4.5 to 5.7 years of accelerated biological aging. Apparently, percent weight gain plays a critical role in the biological aging of U.S. women. Clearly, greater public health efforts are needed to help U.S. women manage their body weight over time. 

## Figures and Tables

**Table 1:** Percentile distribution of the key continuous variables representing U.S. adults (*n* = 3070).

Variable	Percentile				
	10th	25th	50th	75th	90th
Age (years)					
Women	38.1	42.1	49.2	57.2	64.5
Men	37.6	41.0	47.9	56.1	63.7
10-year weight change (%)					
Women	–5.0	3.3	10.4	19.3	27.5
Men	–8.2	–0.8	5.2	11.9	17.9
Telomere length (base pairs)					
Women	5136.3	5385.8	5699.3	6101.3	6594.3
Men	5099.8	5340.9	5650.2	6086.3	6569.4
C-reactive protein (mg/dL)					
Women	0.05	0.11	0.29	0.65	1.17
Men	0.04	0.07	0.18	0.35	0.74

Note. Table values include person-level weighted adjustments based on the sampling methods of HANES, so values represent those of the U.S. adult population.

**Table 2:** Characteristics of the sample based on the categorical variables (*n* = 3070).

Categorical Variable	N	%	SE
**Sex**			
Women	1500	49.9	0.85
Men	1570	50.1	0.85
**Race/Ethnicity**			
Mexican American	180	5.8	0.75
Other Hispanic	205	6.7	1.50
Non-Hispanic Black	317	10.3	1.30
Other race/multiracial	104	3.4	0.65
Non-Hispanic White	2264	73.8	1.97
**Year of Assessment**			
1999–2000	757	24.7	2.08
2001–2002	2313	75.3	2.08
**Housing Status**			
Renting	656	21.4	1.55
Buying	2352	76.6	1.58
Other	62	2.0	0.37
**Smoking (pack-years)**			
None	2375	77.4	1.23
Low	221	7.2	0.64
Moderate	244	7.9	0.60
High	230	7.5	0.82
**Physical Activity (MET minute)**			
None	1545	50.3	2.05
Low	765	24.9	1.17
Moderate/high	760	24.7	1.69
**BMI**			
Normal weight	895	29.1	0.89
Overweight	1106	36.0	1.26
Obese	1069	34.8	1.34
**Intent to Lose Weight**			
No intent	1858	60.5	1.19
Intent to lose weight	1212	39.5	1.19
**Has a Serious Disease**			
No	2461	80.2	1.07
Yes	609	19.8	1.07
**Menopause Status**			
No menses	968	64.5	1.80
Still having periods	532	35.5	1.80
**Estrogen/Progesterone Use**			
Yes	972	64.8	1.73
No	528	35.2	1.73

Note: SE is the standard error of the percentage. The N and % columns refer to the number of subjects and the percentage of participants, respectively, after the NHANES sample weights were applied. Weighted values are more meaningful than unweighted values because they can be generalized to the U.S. adult population. Having a serious disease includes diabetes, cardiovascular disease, and/or cancer.

**Table 3:** The relationship between percent weight change over 10 years, covariates, and telomere length representing U.S. women and men analyzed separately (*n* = 3070).

Predicting Telomere Length	U.S. Women Only (*n* = 1500)			U.S. Men Only (*n* = 1570)		
Predictors/Covariates	Regression Coefficient	F	*p*	Regression Coefficient	F	*p*
**Age** (years)	–11.47	16.6	0.0003	–16.10	86.0	<0.0001
**Race/Ethnicity**		4.0	0.0102		3.7	0.0157
Mexican American	106.48	3.3	0.0779	175.82	7.0	0.0130
Other Hispanic	256.40	15.8	0.0004	235.57	12.3	0.0015
Non-Hispanic Black	111.65	1.9	0.1797	165.82	3.2	0.0835
Other race/multiracial	174.51	3.2	0.0827	207.67	7.1	0.0123
Non-Hispanic White	Reference	-	-	Reference	-	-
**Housing Status**		0.5	0.5973		0.1	0.8941
Buying	−177.37	1.0	0.3264	−57.16	0.1	0.7346
Renting	−157.82	0.8	0.3688	−44.22	0.1	0.8040
Other	Reference	-	-	Reference	-	-
**Year of Assessment**						
1999–2000	−175.34	3.9	0.0575	−39.18	0.3	0.5998
2001–2002	Reference	-	-	Reference	-	-
**Body Mass Index**		0.5	0.6219		4.6	0.0190
Normal weight	−10.58	0.0	0.8415	137.79	8.4	0.0069
Overweight	−35.94	0.8	0.3749	53.18	3.6	0.0663
Obese	Reference	-	-			
**Smoking Pack-Years**		2.0	0.1315		3.1	0.0407
None	165.66	4.8	0.0377	146.29	4.2	0.0490
Low	243.29	5.0	0.0331	55.36	0.4	0.5214
Moderate	282.05	5.3	0.0280	58.20	0.9	0.3586
High	Reference	-	-	Reference	-	-
**Intent to Lose Weight**						
No intent	27.24	0.4	0.5157	86.69	9.6	0.0042
Tried to lose weight	Reference	-	-	Reference	-	-
**MET-Minutes of Activity**		1.5	0.2419		2.6	0.0950
None	−94.59	1.4	0.2520	−30.65	0.2	0.6439
Low	−96.72	2.8	0.1082	−120.89	3.9	0.0569
Moderate/high	Reference	-	-	Reference	-	-
**Has a Serious Disease**						
No	74.69	2.0	0.1721	19.39	0.2	0.6401
Yes	Reference	-	-	Reference	-	-
**C-reactive Protein (mg/dL)**	−38.69	4.7	0.0391	−23.66	1.4	0.2541
**Menopause Status**						
No menses	−39.98	0.9	0.3555	-	-	-
Having periods	Reference	-	-	-	-	-
**Estrogen/Progesterone Use**				-	-	-
Yes	−41.83	0.8	0.3731	-	-	-
No	Reference	-	-	-	-	-
**Percent Weight Change**	−3.48	4.7	0.0392	2.39	1.6	0.2099

Note: Each variable is listed as if it was last in the model, with all the other variables controlled. For the female sample, R^2^ was 11.4% and R^2^ was 10.2% for the male sample. The NHANES clusters, strata, and individual sample weights were applied. Weighted values are more meaningful than unweighted values because they can be generalized to the U.S. adult population. Statistical significance was based on 29 degrees of freedom (57 clusters–28 strata).

## Data Availability

All data supporting reported results can be found online as part of the National Health and Nutrition Examination Survey (NHANES). The data are free and can be found at the following website: https://wwwn.cdc.gov/nchs/nhanes/Default.aspx (accessed on 15 June 2023).
